# Neural Mechanisms Underlying Metacognitive Processes in Sequential Decision-Making

**DOI:** 10.1523/ENEURO.0104-25.2025

**Published:** 2026-10-28

**Authors:** Yoshinori Nanjo, David Aguilar-Lleyda

**Affiliations:** ^1^Department of System Neuroscience, Division of Cerebral Integration, National Institute for Physiological Sciences, Okazaki, Aichi 444-8585, Japan; ^2^Department of Physiological Sciences, School of Life Science, SOKENDAI (The Graduate University for Advanced Studies), Hayama, Kanagawa 240-0193, Japan; ^3^RIKEN CBS-TOYOTA Collaboration Center (BTCC), RIKEN Center for Brain Science, Wako, Saitama 351-0198, Japan

**Keywords:** dynamic causal modeling, fMRI, medial prefrontal cortex, metacognition, sequential decision-making

## Abstract

Self-monitoring in decision-making is a hallmark of self-improvement in cognitive processes. To enhance outcomes in future decisions, individuals must accurately assess the correctness of prior choices and apply the information to control subsequent decisions. Although metacognition encompasses monitoring and control functions, the neural mechanisms underlying these processes and their relationships remain unclear. Furthermore, while the prefrontal cortex (PFC) is implicated in both functions, their distinct neural substrates and interrelations require further elucidation. In this study, we employed a sequential perceptual decision-making task with confidence ratings to examine how initial confidence influences subsequent decisions while recording neural activity using functional magnetic resonance imaging (fMRI). Subjects (female and male) performed a visual discrimination of two different-sized stimuli and subsequently rated their decision confidence. Behavioral results showed that subjects were more likely to change their choices between initial and subsequent decisions according to confidence levels. A signature of confidence (confidence magnitude) in initial decisions was observed primarily in the anterior medial PFC (amPFC). In contrast, the dorsal anterior cingulate cortex (dACC) exhibited higher neural activity related to changes of their initial choice at subsequent decisions. Furthermore, the pregenual ACC (pgACC) was associated with both processes, and it exhibited effective connectivity with the amPFC and dACC. These findings suggest that the medial PFC plays a role in integrating metacognitive monitoring and subsequent control in the sequential decision-making processes.

## Graphical abstract

**Figure:**
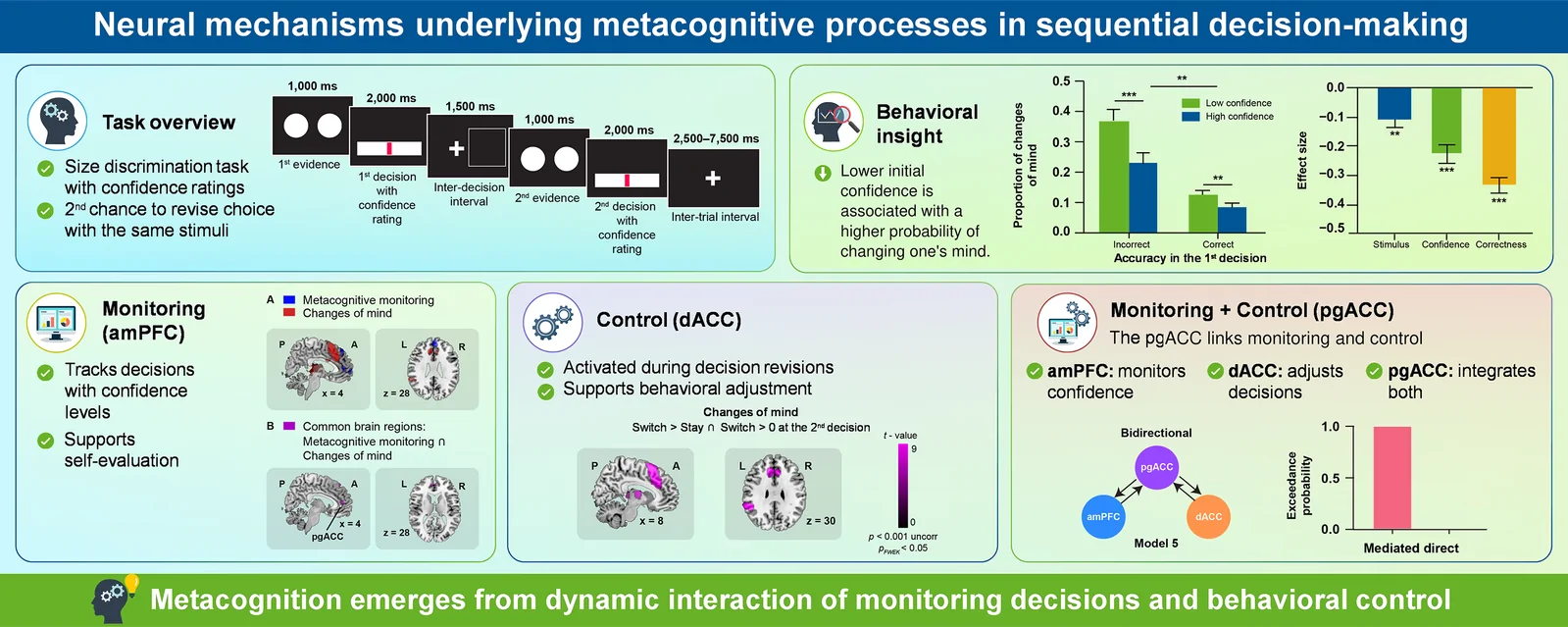
Visual Abstract

## Significance Statement

Metacognition, encompassing monitoring and control, is fundamental to self-regulation and sequential decision-making. Despite their distinct roles, the neural mechanisms of how monitoring connects to subsequent control remain unclear. Using functional magnetic resonance imaging (fMRI), we reveal distinct yet interconnected medial prefrontal regions that support metacognitive processes. Specifically, the anterior medial prefrontal cortex encodes confidence in initial decisions, while the dorsal anterior cingulate cortex supports changes of mind. Effective connectivity analysis indicates that the pregenual anterior cingulate cortex integrates these processes. These findings advance our understanding of medial prefrontal mechanisms underlying adaptive self-regulation, with implications for addressing disorders characterized by impaired behavioral control.

## Introduction

Self-monitoring in decision-making is a hallmark of self-improvement in cognitive processes. Metacognition is a cognitive function that allows us to monitor our cognition and control subsequent behavior and mental states (Flavell, 1979; Nelson and Narens, 1990). Prior research has focused on monitoring processes in metacognition (metacognitive monitoring), measuring the extent to which confidence reflects decision accuracy, often referred to as metacognitive ability (Dunlosky and Lipko, 2007; Mazancieux et al., 2020). Although studies have primarily focused on monitoring processes in perceptual decisions, our daily actions often involve sequences of decisions rather than a single decision (Nelson and Narens, 1990; Kiani et al., 2014; Desender et al., 2018). This focus on metacognitive monitoring alone might overlook the inherent role of metacognition in subsequent control. We confirmed that confidence in initial decisions influenced subsequent changes of mind independently of perceptual evidence. Consistent with these behavioral findings, we found that medial frontal regions were associated with these metacognitive processes.

The prefrontal cortex (PFC) has been thought to play an integrative role in metacognitive processes and cognitive control at the object level. Neuroimaging studies have linked the lateral PFC to individual differences in metacognitive ability in perceptual and memory-based decision-making (Fleming et al., 2010, 2012; McCurdy et al., 2013; Morales et al., 2018). However, earlier studies have also implicated the medial PFC in aspects of metacognition closely related to executive functions (Fernandez-Duque et al., 2000; Shimamura, 2000). Furthermore, the neural mechanisms underlying cognitive (behavioral) control have also been investigated. The dorsal anterior cingulate cortex (dACC) in the medial frontal regions (Johansen-Berg et al., 2004; Beckmann et al., 2009; Vogt, 2009) is related to error/conflict monitoring and executive functions (Botvinick et al., 2001; Shenhav et al., 2016; Musslick and Cohen, 2021; Mansouri et al., 2022). Together, these findings suggest that the medial PFC contributes to control over cognition and to the implementation of such control in temporally extended contexts. Damage to the PFC due to disease or injury can result in a dissociation of these functions, as observed in dysexecutive syndromes (Damasio and Anderson, 1993). Hence, we hypothesized that the neural substrates underlying metacognitive monitoring and control would be spatially dissociated.

However, these metacognitive processes are closely associated with each other. Previous studies examining metacognition in sequential contexts have begun to elucidate the role of metacognition in recognizing and rectifying errors in decision-making (Butterfield and Mangels, 2003; Yeung and Summerfield, 2012; Boldt and Yeung, 2015; Kononowicz and van Wassenhove, 2019). Despite these advances, only a few studies have explicitly examined both metacognitive processes (Qiu et al., 2018; Boldt and Gilbert, 2022). Hence, the relationship between metacognitive monitoring and subsequent control processes has not been investigated independently of external evidence. To address this gap, experimental paradigms should include behavioral measures of both monitoring and control in sequential contexts.

In the present study, we designed a sequential perceptual decision-making task with confidence ratings to investigate how confidence in initial decisions influences subsequent decision-making independently of external evidence. We investigated the neural mechanisms linking metacognitive monitoring to subsequent control while recording markers of neural activity in the human brain using functional magnetic resonance imaging (fMRI). We hypothesized that distinct regions within the medial PFC, including the dACC, would be involved in metacognitive monitoring and subsequent control. This study addressed the following issues: (1) the role of metacognitive monitoring in influencing subsequent decision-making, (2) neural representations of metacognitive monitoring in the PFC, (3) neural substrates of subsequent control processes, and (4) neural interaction between these processes.

## Materials and Methods

### Subjects

This study was conducted with 34 right-handed Japanese general volunteers [24 females and 10 males, age: 23.56 ± 3.47 years (mean ± SD), ranging from 20 to 34 years]. The number of subjects is consistent with recent criteria for the sample size of a neuroimaging study (Poldrack et al., 2017). All the subjects reported normal visual acuity and no history of major medical or neurological illnesses. Written informed consent was obtained from all subjects in accordance with the Declaration of Helsinki. The local medical ethics committee approved the experimental procedures at the National Institute for Physiological Sciences. In the fMRI analyses, we excluded two subjects (one female and one male) due to the unavailability of their T2-weighted images, which prevented us from performing the same preprocessing as was conducted for the other subjects (Glasser et al., 2013; Smith et al., 2013).

### Experimental setup, task design, and procedures

The experimental task was implemented using Psychtoolbox-3 in MATLAB R2018b (MathWorks). We equidistantly presented visual stimuli consisting of two white circles on a black background on a screen using a projector (resolution, 1,280 × 1,024; distance, 190.8 cm; visual angle, 1.02°). Subjects viewed the screen through a mirror. The half-transparent viewing screen was located behind the MRI head coil, and visual cues were projected using a liquid-crystal display projector. Subjects held an optical response button box (HHSC1×4-D; Current Designs) in their right hand to record their behavioral responses. During the intertrial intervals, the subjects were instructed to look at a white crosshair placed at the center of the screen. Before performing the main experiment, the subjects practiced the experimental task to familiarize themselves with the task and general procedures inside the scanner for ∼2 min.

We designed and conducted a novel experimental task using a two-alternative forced choice (2AFC) paradigm, which involved sequential perceptual decision-making with confidence ratings (Fig. 1*A*). This experimental task was designed to investigate how confidence in initial decisions influences subsequent decision-making, specifically whether subjects choose to switch from their initial decision (changes of mind). Each trial consisted of the following five phases: (1) presentation of the first stimulus (first evidence); (2) first decision and confidence ratings; (3) interdecision interval; (4) presentation of the second stimulus (second evidence); and (5) second decision and confidence ratings. Within a single trial, each subject viewed two circular stimuli on the left and right sides of the center of the screen for 1 s at the time of the presentation of the first and second stimuli. Circular stimuli were presented at four levels of size difference (0.75, 1.5, 3.0, and 6.0% difference). The same stimulus set was presented at both stimulus presentations. In each decision and confidence rating phase, subjects were required to respond with a button press to indicate which circular stimulus they perceived as larger and rate their confidence on a fifty-point scale (1 = minimum, 50 = maximum confidence) using a visual analog scale. In terms of confidence ratings, we instructed them to report the relative confidence in their performance in this task. Each subject made their first decision and provided confidence ratings within 2 s. The first decision was followed by an interdecision interval of 1.5 s, during which the first decision was displayed with the marker positioned either on the left or right side of the center of the screen, according to the initial choice. Subjects then viewed the same stimulus set again for 1 s before making their second decision and providing confidence ratings within 2 s. After the second decision and confidence ratings, a white crosshair was presented during an intertrial interval (2.5 or 7.5 s).

The current experiment used an event-related fMRI design consisting of four scanning runs, each comprising 48 trials [4 stimulus differences × 2 locations of the larger stimulus (left or right) × 2 experimental conditions × 3 repetitions]. Each run lasted ∼8 min and 40 s. The experimental recordings were ∼50 min in total. To improve the efficiency of the fMRI data, we utilized a genetic algorithm (Wager and Nichols, 2003) to arrange the order of each experimental condition and stimulus set with different stimulus sizes across trials. Within each run, jittering intertrial intervals were set at either 2.5 or 7.5 s, and the fixation crosshair was displayed for the first and last 10 s of each run. Consequently, four trial-order sequences were created and randomly assigned to the four scanning runs.

Another experimental condition was simultaneously conducted using two MRI scanners. In half of the trials, only the subjects' own first decision was presented, while in the other half, the first decision of another subject performing the same task was displayed during an interdecision interval. In the present study, we focused only on the experimental condition displaying a self-decision during an interdecision interval to investigate the neural mechanisms underlying metacognitive processes within individuals.

### Behavioral data analysis

#### Psychometric function fitting

Perceptual decision-making is based on external evidence (Gold and Shadlen, 2007). To examine whether the subjects based their decisions on external evidence and engaged in perceptual decision-making during the task, the sensitivity of the psychometric curves was estimated. Psychometric functions were constructed by fitting the proportion of trials where the right stimulus was chosen as the larger stimulus (*y*-axis) against the stimulus difference levels (*x*-axis; a positive value indicated that the right stimulus was larger). We fitted the psychometric function using a logistic function (Fig. 1*B*). To estimate the perceptual sensitivity parameters, a model was employed using the following equation:

$$p=\;\frac{1}{1\;+\exp\left(-s(x-x_{0})\right)},$$

where *p* is the proportion of right responses, *x* is the size difference between the right and left stimuli, *x*_0_ is the indifference point (the size difference of the stimuli such that the proportion of choosing the right side is the same as the proportion of choosing the left side), and *s* is a parameter controlling the steepness of the psychometric function, with larger values indicating a steeper slope. Therefore, *s* reflects the sensitivity of size discrimination. We compared the slope of each decision (Fig. 1*C*) to determine whether (1) each slope was positive and (2) the slope of the second decision was steeper than that of the first decision using one-tailed one-sample and two-tailed paired *t* tests, respectively.

#### Confidence rating

To examine whether subjective confidence was influenced by evidence, we tested the effects of the stimulus size differences on confidence ratings at each decision and compared confidence across decisions (Fig. 1*D*). The confidence scores of the subjects were averaged across trials for each stimulus size difference for each decision. We conducted a two-way repeated-measures ANOVA, assessing within-subject factors of stimulus size difference (0.75, 1.5, 3.0, and 6.0% difference) and decisions (first and second decision). We applied the Greenhouse–Geisser correction when the assumption of sphericity was violated, as determined by Mauchly's test of sphericity results.

#### Changes of mind

The proportion of changes of mind was examined to investigate how confidence in initial decisions influences subsequent decision-making (Fig. 2). All trials were split into high and low confidence trials based on the median of initial confidence within each stimulus size difference. Then, the proportion of changes of mind (first and second decisions being different) was calculated. This allowed us to examine confidence in the initial decision while controlling for the influence of external factors, such as stimulus size differences, on subjective confidence. To analyze the changes of mind, we performed a two-tailed paired *t* test between the high and low confidence trials. We also investigated the role of confidence in error detection, as confidence has been shown to play a functional role in error detection (Butterfield and Mangels, 2003; Yeung and Summerfield, 2012; Boldt and Yeung, 2015; Harty et al., 2017; Kononowicz and van Wassenhove, 2019). The proportion of changes of mind was calculated for trials where the initial decision was correct or incorrect, given high and low confidence trials in initial decisions. We then performed a two-way repeated-measures ANOVA, assuming within-subject factors of initial confidence (high and low confidence) and initial responses (correct and incorrect responses). We applied the Greenhouse–Geisser correction for the cases where the results of Mauchly's test of sphericity were significant.

#### Analysis of changes of mind with a mixed-effects logistic regression

We also analyzed the influence of initial confidence on changes of mind using a mixed-effects logistic regression with the lme4 package in R version 4.4.3. The changes of mind in trial *t* of subject *j* were modeled as follows:

$$p_{tj}=\;\frac{1}{1+\exp(-\eta_{tj})}\;,$$

$$\mathrm{Model}\;1:\;\eta_{tj}=\beta_{0}+\beta_{1}\mathrm{con}f_{tj}+\beta_{2}\mathrm{sti}m_{tj}+\beta_{3}\mathrm{cor}r_{tj}+u_{0j},\;$$

$$u_{0j}\;∼\;N(0,\;\sigma_{u0}^{2}),$$

where conf is the confidence at the first decision, stim is the size difference between the two stimuli, and corr is the correctness of the first decision. Both confidence and stimulus difference were *z*-scored, with *β* representing the fixed-effect coefficient for each variable, *β*_0_ as the fixed-effect intercept, and *u*_0*j*_ as the subject-specific random intercept. Subjects were treated as random effects, and the probability of changes of mind was estimated as *p* (switch = 1, stay = 0). This model can be considered a main effects model with a random intercept. We then performed model comparison using the Bayesian information criterion (BIC), with lower values indicating a preferred model (Schwarz, 1978).

$$\mathrm{Model}\;2:\;\eta_{tj}=\beta_{0}+\beta_{1}\mathrm{con}f_{tj}+\beta_{2}\mathrm{sti}m_{tj}+\beta_{3}\mathrm{cor}r_{tj}+u_{0j}+u_{1j}\mathrm{con}f_{tj}+u_{2j}\mathrm{cor}r_{tj},$$

$$\left(\begin{array}{c} u_{0j}\\ u_{1j}\\ u_{2j} \end{array}\right)\;∼\;\mathrm{MVN}(0,\;\Sigma ).$$

Building on Model 1, we introduced subject-specific slopes for confidence (*u*_1_) and correctness (*u*_2_) to capture individual variability in their effects, while retaining a random intercept. No random slope was specified for stimulus difference. The variance-covariance matrix Σ describes the extent and covariation of these subject-level deviations.

$$\mathrm{Model}\;\;3:\;\eta_{tj}=\beta_{0}+\beta_{1}\mathrm{con}f_{tj}+\beta_{2}\mathrm{cor}r_{tj}+\beta_{3}(\mathrm{con}f_{tj}\times \mathrm{cor}r_{tj})+\beta_{4}\mathrm{sti}m_{tj}+u_{0j},$$

$$u_{0j}\;∼\;N(0,\;\sigma_{u0}^{2}).$$

Model 3 extends the main effects specification (Model 1) by adding a fixed-effects interaction between confidence and correctness to test whether confidence influences changes of mind differently on correct versus incorrect trials while controlling for stimulus differences.

$$\begin{array}{rl} \mathrm{Model}\;\;4:\;\eta_{tj} & =\beta_{0}+\beta_{1}\mathrm{con}f_{tj}+\beta_{2}\mathrm{cor}r_{tj}+\beta_{3}(\mathrm{con}f_{tj}\times \mathrm{cor}r_{tj})+\beta_{4}\mathrm{sti}m_{tj}+u_{0j}+u_{1j}\mathrm{con}f_{tj}\\ & \;+u_{2j}\mathrm{cor}r_{tj}+u_{3j}(\mathrm{con}f_{tj}\times \mathrm{cor}r_{tj}), \end{array}$$

$$\left(\begin{array}{c} u_{0j}\\ u_{1j}\\ u_{2j}\\ u_{3j} \end{array}\right)\;∼\;\mathrm{MVN}(0,\;\Sigma ).$$

To explain individual differences not only in overall decision tendencies but also in how confidence, correctness, and their interaction influence changes of mind, we added random slopes to Model 3. The variance-covariance matrix Σ captures the variances of and covariances among the random intercept (*u*_0*j*_), the random slopes for confidence (*u*_1*j*_) and correctness (*u*_2*j*_), and the random slope for their interaction (*u*_3*j*_).

### MRI data acquisition

We acquired MR images of each subject using a 3 T MRI scanner (Magnetom Verio 3T; Siemens Healthineers) with a standard 32-channel phased-array coil. Whole-brain functional images were obtained using multiband gradient-echo echoplanar imaging (EPI) sequences (TR, 1,000 ms; TE, 35 ms; flip angle, 65°; FOV, 192 × 192 mm^2^; matrix size, 96 × 96; slice thickness, 2.5 mm; 60 transverse slices; voxel size, 2.0 × 2.0 × 2.5 mm^3^; multiband acceleration factor, 6; phase-encoding direction, anterior to posterior). Each run consisted of 520 volumes, and a total of four runs were performed. Before the main functional scans, spin-echo EPI images were acquired with the opposite phase-encoding direction for subsequent susceptibility distortion correction in the preprocessing of functional images (TR, 7,560 ms; TE, 64 ms; in-plane FOV, 192 × 192 mm^2^; matrix size, 96 × 96; 60 transverse slices; FA, 90°; refocus FA, 180°; BW, 1,860 Hz/pixel; and ES, 0.76 ms). In another session, T1- and T2-weighted structural images with 0.8 mm isotropic resolution were obtained, as in the previous study (Yamamoto et al., 2021).

### MRI data analyses

#### Preprocessing

We used the Human Connectome Project (HCP) Pipelines v4.0.1 (Glasser et al., 2013) for MRI data preprocessing to correct for gradient nonlinearity-induced distortion, head motion, and susceptibility-induced distortion and to perform normalization, as in previous studies (Glasser et al., 2013, 2016; Yamamoto et al., 2021). We applied FMRIB's ICA-based Xnoiseifier (FIX) to multiple-run-concatenated fMRI time series data (Multirun FIX). This procedure effectively extracted and removed structured noise components observed throughout each subject's session and was implemented as described in previous studies (Smith et al., 2013; Glasser et al., 2016). Prior to concatenation, time series data of each run were demeaned and variance-normalized and temporally high-pass filtered with a full-width at half maximum (FWHM) period of 2,000 s. Then, the normalized time series were concatenated across runs. Melodic ICA implemented in FSL (v6.0.1, Centre for Functional MRI of the Brain, Oxford University, UK; https://fsl.fmrib.ox.ac.uk/fsl/fslwiki/FslInstallation) was applied to the concatenated time series data to produce component spatial maps and time series. Using a classifier trained with the resting-state fMRI data of HCP, these components were automatically classified into signal or noise (Griffanti et al., 2014). The noise components were nonaggressively regressed out of the EPI time series (Glasser et al., 2016). After denoising, the time series data were split back into individual runs, and the spatial mean and variance profiles were restored to the individual runs. We then performed spatial smoothing to improve the signal-to-noise ratio using an isotropic Gaussian kernel with an FWHM of 6 mm, implemented using FSL v6.0.1.

#### Analysis of neural activity

We used the Statistical Parametric Mapping (SPM) 12 software (Wellcome Trust Centre for Neuroimaging, University College London, UK; https://www.fil.ion.ucl.ac.uk/spm/software/download/) implemented in MATLAB R2018b. We performed general linear model (GLM) analysis to assess neural activity related to metacognitive processes at each subject level. The GLM included a regressor covering the first decision and confidence rating period and event regressors associated with switching and staying decisions at the second decision, all convolved with a canonical hemodynamic response function (HRF). The *z*-scored initial confidence and stimulus differences were entered into the GLM as positive parametric modulators for the first decision and confidence rating. This GLM also included regressors for conditions with the presentation of others' initial decisions in the interdecision interval, and temporal derivatives of each regressor were also included in the design matrix as nuisance regressors. The data were high-pass filtered with a cutoff period of 128 s to remove low-frequency drifts, and a global autocorrelation model (AR1) was applied across the whole brain. Parameter estimates were obtained using least-squares estimation.

At the individual level, parameter estimates were obtained using a fixed-effects model, and contrast images were generated for each subject. These contrast images were then entered into group-level random-effects analyses using one-sample *t* tests. We performed these group-level analyses for the parametric modulation of initial confidence and for the contrast between switch and stay events. To examine the positive effects of neural activity related to changes of mind, we performed a conjunction analysis of changes of mind (switch > stay) with the baseline activity of switch trials (switch > 0). In these group-level analyses, we performed probabilistic threshold-free cluster enhancement (pTFCE) on group-level data (Smith and Nichols, 2009; Spisák et al., 2019). We then performed a conjunction analysis to identify common brain regions between contrast images of initial confidence and changes of mind (Nichols et al., 2005).

The statistical threshold for neuroimaging data analyses was set at *p* < 0.05 with familywise error (FWE) correction at the cluster level for the whole brain, with a height threshold of *p* < 0.001 for controlling false positives in all fMRI data analyses (Eklund et al., 2016; Flandin and Friston, 2019). To label brain regions, we used SPM-Anatomy Toolbox v3 (Eickhoff et al., 2007), automated anatomical labeling 3 (Rolls et al., 2020), and the Mai atlas (Mai et al., 2015).

#### Stochastic dynamic causal modeling

Stochastic dynamic causal modeling (sDCM), as implemented in the DCM12.5 add-on for SPM12, was employed to estimate effective connectivity (Friston et al., 2003; Daunizeau et al., 2012; Zeidman et al., 2019). Unlike deterministic DCM, which assumes purely deterministic hidden states, sDCM extends the classical framework by including random fluctuations (noise variance) in the differential equations governing neuronal dynamics (Daunizeau et al., 2012). In sDCM for fMRI, the evolution of hidden neuronal states *x* is described by a stochastic differential equation of the form:

$$\frac{dx}{dt}=Ax+Cu+\omega ,$$

where *A* quantifies intrinsic (endogenous) connections among regions, *C* quantifies the influence of the task-related driving input *u* on neuronal states, and ω represents random perturbations of neural activity (Friston et al., 2003; Daunizeau et al., 2012). By modeling ω, sDCM treats each driving input as a prior on hidden causes, thereby accounting for uncertainty in how exogenous events influence neuronal states (Daunizeau et al., 2012). Although the DCM framework permits modulation of intrinsic connections via *B* matrices and nonlinear gating via *D* matrices (Friston et al., 2003; Stephan et al., 2008), our models omitted both *B* and *D* terms—only *A* and *C* matrices were specified. One event regressor representing the first decision and confidence rating phase was used as the driving input (*C* matrix). The two parametric modulators from the first-level GLM—stimulus difference and confidence—were used to estimate neural time series but were not included in the *C* matrix. Each model was then inverted using the stochastic estimation routine in DCM12.5 (Zeidman et al., 2019). The sDCM formulation yields more accurate parameter and structure inference than deterministic DCM (Daunizeau et al., 2012), providing a principled foundation for characterizing task-related effective connectivity and guiding the subsequent definition of our regions of interest (ROIs).

To examine the relationship between regions associated with metacognitive processes in the medial frontal areas, we defined ROIs based on fMRI results. We identified the peak coordinates corresponding to neural substrates of metacognitive processes: the anterior medial prefrontal cortex (amPFC), dorsal anterior cingulate cortex (dACC), and pregenual anterior cingulate cortex (pgACC). The BOLD time series from separate ROIs were extracted from spheres (radius, 3.0 mm) centered on the MNI coordinates of each region for both hemispheres [left: amPFC (−1, 50, 26), dACC (−10, 30, 30), and pgACC (−3, 45, 30); right: amPFC (1, 50, 26), dACC (10, 30, 30), and pgACC (3, 45, 30)]. To minimize confounding influences, we applied nuisance regression to each ROI time series, regressing out the regressors of no interest, while excluding the regressors of interest on the first decision and confidence rating, the parametric modulator of confidence, and the event regressors of switch and stay.

We specified the endogenous connections (*A* matrix in the DCM) using different connectivity structures—namely, varying directionality (forward, backward, and bidirectional) and mediation (mediated and direct)—resulting in six models in each hemisphere (12 models in total). In the mediated models, we assumed connectivity among the amPFC, pgACC, and dACC, forming a serial pathway (amPFC–pgACC–dACC). In contrast, the direct models included endogenous connectivity only between the amPFC and dACC (the pgACC remained as a node but carried no intrinsic connections). At the group level, we performed random-effects Bayesian model selection (BMS) to explore the most likely structure across subjects. Specifically, all models were first inverted for each subject, yielding model evidence estimates. We then applied a random-effects BMS to predefined model families (Penny et al., 2010; Stephan et al., 2010) based on mediation, directionality, and laterality. Family-level BMS aggregates evidence across models, yielding a posterior probability for each family. By applying random-effects BMS at the family level, we ensured that our inferences of mediation, directionality, and laterality were robust to intersubject variability and focused on the most plausible network configurations.

## Results

### Basic behavioral results

The basic behavioral analyses were first conducted to confirm whether the subjects' behavior in response to external evidence was as required. In this experiment, evidence for perceptual decisions originated from the size differences between two circular stimuli, which were presented twice as a pair in a single trial (Fig. 1*A*).

**Figure 1.**
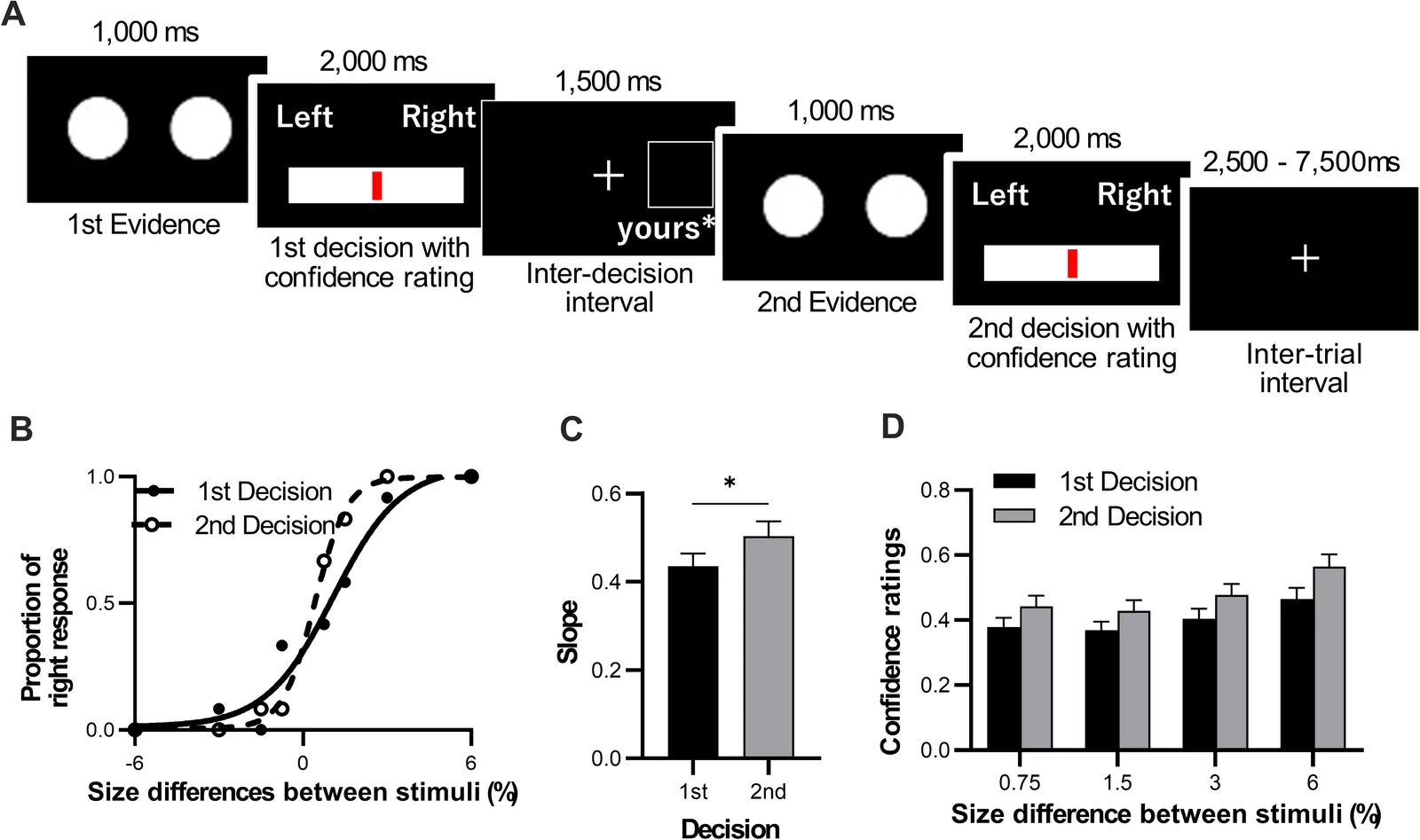
Experimental task and basic behavioral results. ***A***, Illustration of the experimental task in one trial. Subjects were required to discriminate which circular stimulus was larger and rate their confidence. Each subject viewed the same stimulus during the first and second evidence presentations. After each decision, no feedback on the outcome of their decision was provided. For clarity, only the solo condition, in which subjects viewed their own initial decision during the interdecision interval, is illustrated. ***B***, Psychometric function for a representative subject. The *x*-axis represents the standardized size difference between the right and left stimuli. The *y*-axis is a proportion of right responses. The solid line shows the fitted psychometric curve of the first decision, and the dashed line is that of the second decision. The steepness of the slopes of the psychometric curves reflects the sensitivity to the size contrast between the two circular stimuli. ***C***, Mean of psychometric slopes across subjects at each decision timing. The slope of the second decision was steeper than that of the first decision. The error bars are the SEM. “*” is *p* < 0.05. ***D***, Mean of subjective confidence across stimulus differences for the first and second decisions. This panel shows subjective confidence for stimulus differences and each decision. Each bar shows the average value of confidence rating scores across subjects. The *x*-axis represents the stimulus difference (%), and the *y*-axis is the standardized confidence. The error bars indicate SEM.

The psychometric functions were fitted to behavioral data to examine (1) whether the trends of these psychometric slopes were positive (indicating a higher likelihood of choosing the right stimulus when the size difference between the stimuli increases) and (2) whether the repeated presentation of the stimuli enhanced discrimination accuracy of perceptual decisions (higher accuracy in the second decision when compared with that of the first decision). A psychometric function for each decision was fitted based on maximum likelihood estimation, and slopes were estimated (Fig. 1*B*,*C*). The results from the one-tailed one-sample *t* test confirmed that the slopes of the first and second decisions were positive (the first decision: 0.436 ± 0.029 [mean ± standard error of the mean (SEM)]; *t*_(33)_ = 15.28, *p* < 0.001; the second decision: 0.503 ± 0.033 (mean ± SEM); *t*_(33)_ = 15.15, *p* < 0.001). These results showed that the proportion of the right responses increased as the right stimulus became relatively larger than the left stimulus. Subsequently, we compared these slopes using a two-tailed paired *t* test and found that the slope of the second decision was significantly steeper than that of the first decision (*t*_(33)_ = 2.60, *p* = 0.014). This suggests that evidence from external stimuli enhanced the accuracy of perceptual decisions (Shadlen and Newsome, 2001; Gold and Shadlen, 2007; Murphy et al., 2015; Rangelov and Mattingley, 2020).

Stimulus evidence influences not only perceptual decisions but also subjective confidence (Koriat, 2011, 2012; Murphy et al., 2015). In this framework, repeated presentation of the same stimulus makes their responses more accurate (Shadlen and Newsome, 2001; Gold and Shadlen, 2007) and also enhances confidence in perceptual decisions (Kiani and Shadlen, 2009; Lebreton et al., 2015). We performed a two-way repeated-measures ANOVA with within-subject factors of stimulus difference (0.75, 1.5, 3.0, and 6.0% difference) and decision (the first and second decisions) to examine whether subjective confidence was influenced by accumulation of evidence (Fig. 1*D*). To assess the influences of evidence on subjective confidence within a single decision, we confirmed the main effect of stimulus difference (*F*_(3,99)_ = 22.18, *p* < 0.001). The main effect of decision was also significant (*F*_(1,33)_ = 19.61, *p* < 0.001). However, the interaction between the two factors did not reach significance (*F*_(3,99)_ = 2.36, *p* = 0.076).

Overall, these results suggest that the subjects in this study performed well as required in this experimental task.

### Changes of mind

In this experimental task, the external stimulus influenced subjective confidence. However, it is also possible that metacognitive processes encompass internal components that operate independently of these external influences. For instance, confidence is also known to contribute to confirmation bias and error detection (Nickerson, 1998; Yeung and Summerfield, 2012). Here, to investigate the influences of initial confidence on subsequent decision-making, we split all trials for the first decision into high and low confidence trials based on the median of initial confidence in each stimulus difference, and the distinction between them did not rely on the external stimulus. First, we examined whether initial confidence influences subsequent decision-making by comparing the proportion of changes of mind from the first to the second decision. We observed that the proportion of changes of mind in the high confidence trials was significantly lower than that of low confidence trials [high confidence trials: 0.120 ± 0.019 (mean ± SEM); low confidence trials: 0.205 ± 0.019 (mean ± SEM); *t*_(33)_ = −4.59, *p* < 0.001; Fig. 2*A*].

**Figure 2.**
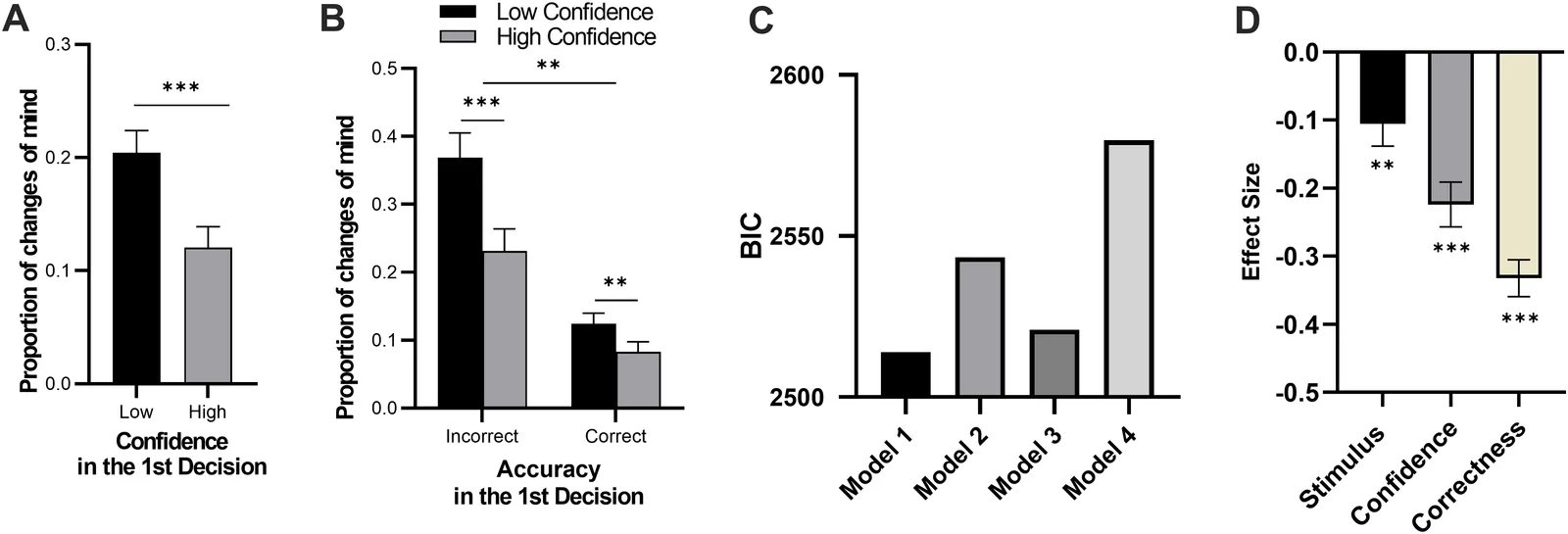
Effects of confidence on changes of mind. ***A***, This figure shows the proportion of changes of mind from the first to the second decisions (*y*-axis) for high and low confidence trials (*x*-axis). The error bars represent SEM. “***” represents *p* < 0.001. ***B***, This graph includes information on the initial response (correct/incorrect) given the initial confidence. This allows us to examine whether initial confidence affects the changes of mind between correct and incorrect responses. The error bars represent SEM. “***” is *p* < 0.001, and “**” is *p* < 0.01. ***C***, BIC results across models using a mixed-effects logistic regression. ***D***, This graph shows the result of a mixed-effects logistic regression analysis. The explanatory variables for the proportion of changes of mind are confidence, stimulus, and correctness (“**” *p* < 0.01, and “***” *p* < 0.001). Fixed-effect coefficients were standardized by multiplying each coefficient by the SD of its predictor across all analyzed trials and dividing by *π*/√3, the SD of the logistic error distribution. Error bars indicate SEs rescaled by the same factors. The results report the model coefficients and SEs before this rescaling. Control analyses addressing multicollinearity are provided in Extended Data Figures 2-1 and 2-2: the predictor correlation matrix and variance inflation factors (VIF), respectively.

10.1523/ENEURO.0104-25.2025.f2-1 Figure 2-1 **Correlation matrix among confidence, stimulus, and correctness** To assess multicollinearity in our behavioral data analyses on changes of mind, this figure displays correlation coefficients among confidence, stimulus, and correctness. All off-diagonal correlations were less than 0.30, indicating negligible collinearity. Download Figure 2-1, DOCX file.

10.1523/ENEURO.0104-25.2025.f2-2 Figure 2-2 **VIF in behavioral data analyses** To assess the degree of multicollinearity, we calculated the variance inflation factor (VIF). This figure shows that all VIFs are below 5.00, indicating that multicollinearity is negligible. Download Figure 2-2, DOCX file.

Metacognition influences the processes of error detection and correction (Yeung and Summerfield, 2012). Thus, the observed effects of confidence on subsequent decisions might instead reflect the impact of error detection. We performed a two-way repeated-measures ANOVA with within-subject factors: initial confidence (high and low) and initial response (correct and incorrect) to examine whether initial confidence influenced subsequent decisions independently of the effects of error detection and correction (Fig. 2*B*). The main effects of initial confidence and initial response were significant (initial confidence: *F*_(1,33)_ = 19.54, *p* < 0.001; initial response: *F*_(1,33)_ = 66.02, *p* < 0.001). There was a significant interaction between them (*F*_(1,33)_ = 10.72, *p* = 0.002). These results suggest that initial confidence influences subsequent decision-making independently of the objective accuracy.

Subsequently, we conducted a mixed-effects logistic regression analysis to examine whether initial confidence influenced changes of mind independently of external stimuli and initial responses. The results demonstrated that Model 1 (main effects model with a random intercept) had a lower BIC than the other three models (Models 2–4; Model 1: 2,513.9, Model 2: 2,543.3, Model 3: 2,520.9, Model 4: 2,579.6; Fig. 2*C*). Model 1 showed that initial confidence, stimulus differences, and initial correctness contributed to determining whether to switch from or stay with their initial decisions (initial confidence: *β*_1_ = −0.408, se = 0.060, *p* < 0.001; stimulus differences: *β*_2_ = −0.221, se = 0.068, *p* = 0.001; correctness: *β*_3_ = −1.340, se = 0.109, *p* < 0.001; Fig. 2*D*). The estimated standard deviation of the subject-random intercept was 0.782 (variance = 0.611), indicating substantial between-subject heterogeneity in the baseline proportion of changes of mind. To confirm the robustness of this analysis, we assessed multicollinearity in the fixed-effect predictors and observed no problematic intercorrelations (Extended Data Figs. 2-1, 2-2). This result also revealed that initial confidence influenced changes of mind independently of stimulus differences and initial responses; therefore, we considered the behavior in this task to be a part of metacognitive processes.

### Neural activity associated with confidence in initial decisions

We confirmed that confidence in initial decisions played a role in modulating the proportion of changing and sustaining choices across decisions (Fig. 2). To examine neural substrates linking the monitoring of the initial decisions to the subsequent control processes, we identified the brain regions responsible for metacognitive monitoring.

We performed fMRI data analysis, comparing behaviorally identified variables against the BOLD signals recorded across the whole brain. To investigate metacognitive monitoring-related neural activity, we first analyzed neural activity during the first decision and confidence rating period using the parametric modulators of initial confidence and stimulus differences. The significant neural activity related to confidence ratings in initial decisions was observed in the anterior medial prefrontal cortex (amPFC) and inferior frontal gyrus (IFG), whereas the neural activity for stimulus differences was not found when these parametric modulators were compared with baseline (*p* < 0.05 FWE corrected at the cluster level with a height threshold of *p* < 0.001; Fig. 3, Table 1). Given that confidence ratings are influenced not only by stimulus differences but also by response times, attributing our findings solely to confidence-related activity is not justified. To corroborate our findings, we therefore included response time as an additional parametric modulator and estimated whether the original activation patterns persisted. This control analysis demonstrated that the regions identified here remained distinct from those explained by response time (Extended Data Figs. 3-1, 3-2). Although the brain regions sensitive to the confidence ratings during the first decision were identified, it is necessary to investigate the subsequent neural processes where changes of mind occur to examine the neural substrates of subsequent control processes.

**Figure 3.**
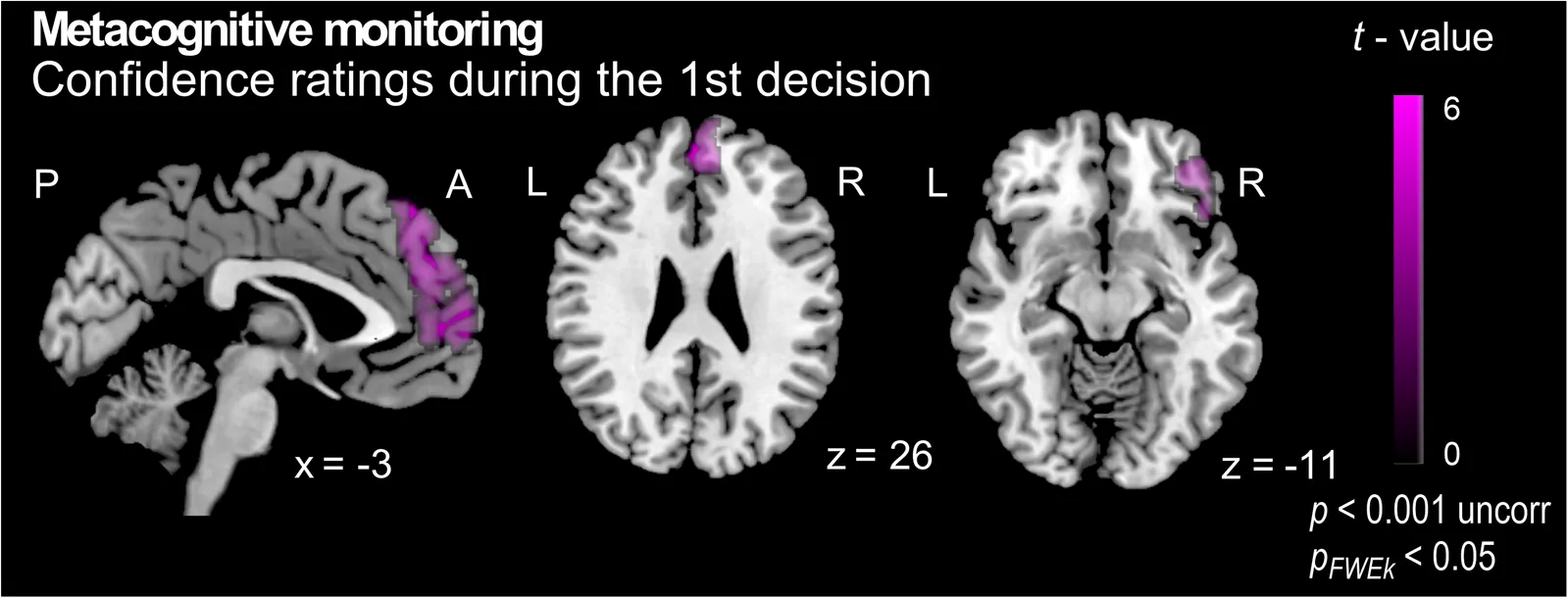
Neural activity associated with metacognitive monitoring. This figure displays group-level statistical maps, depicting neural activity of metacognitive monitoring. To identify the brain regions sensitive to confidence in initial decisions, we analyzed neural activation during the first decision using the parametric modulation of initial confidence and stimulus differences for each subject. A random-effects model was used for group-level analysis (significance set at *p* < 0.05, FWE corrected at the cluster level with a height threshold of *p* < 0.001). The color bar represents the color coding of the *t* value on the statistical map. The “*x*” beside the sagittal slice and “*z*” beside the axial slice denote the x- and *z*-coordinates of the displayed brain slices in MNI space, respectively. The abbreviations used here are as follows: P for posterior, A for anterior, L for left, and R for right. Control analyses for neural activity of metacognitive monitoring are provided in Extended Data Figures 3-1 and 3-2.

10.1523/ENEURO.0104-25.2025.f3-1 Figure 3-1 **Neural substrates of metacognitive monitoring in GLM2** To examine neural activity of confidence in initial decisions, we analyzed fMRI data using a period of the first decision and confidence rating with parametric modulators of initial confidence and stimulus differences (see Fig. 3 and Table 1). However, confidence is influenced by stimulus differences as well as response time. To corroborate our main findings, we analyzed neural activation of confidence in initial decisions using another GLM (GLM2), which added a parametric modulator of response time in initial decisions into the main GLM. In this analysis, we found similar activity patterns (significance set at *p* < 0.05, FWE corrected at the cluster level with a height threshold of *p* < 0.001). The color bar represents the color coding of the t-value on the statistical map. The “x” beside the sagittal slice and “z” beside the axial slice denote the x- and z-coordinates of the displayed brain slices in MNI space, respectively. The abbreviations used here are as follows: P for posterior, A for anterior, L for left, and R for right. Download Figure 3-1, TIF file.

10.1523/ENEURO.0104-25.2025.f3-2 Figure 3-2 MNI coordinates of neural substrates of initial confidence in GLM2 Download Figure 3-2, DOCX file.

**Table 1.** MNI coordinates of neural substrates of metacognitive monitoring

Cluster size (mm^3^)	MNI coordinates				Peak value		Region label
	*x*	*y*	*z*	L/R	*t* value	*p* value FWE-corr	
12,320	−1	50	26	L	5.93	0.100	Anterior medial prefrontal cortex
	−3	45	41	L	5.53	0.233	Anterior medial prefrontal cortex
	−14	39	54	L	5.41	0.301	Superior frontal gyrus
3,380	−38	41	−11	L	4.75	0.801	Inferior frontal gyrus, pars triangularis
	−47	26	−9	L	4.74	0.804	Inferior frontal gyrus, pars triangularis
	−51	28	4	L	4.42	0.964	Inferior frontal gyrus, pars triangularis

### Neural substrates of changes of mind

The primary interest of this study was to identify the neural substrate of metacognitive monitoring, which we found to reside in the amPFC at the within-subject level. We then analyzed fMRI data to examine the brain regions involved in the changes of mind at the second decision.

With the same GLM used for analyzing initial confidence, we analyzed neural activity of changes of mind, comparing neural activity of switch trials with that of stay trials. The analysis of the contrast between switch and stay with the positive activation of switch > baseline revealed significantly higher activity primarily in the dorsal anterior cingulate cortex (dACC), inferior parietal lobule (IPL), IFG, and anterior insular cortex (aIC; *p* < 0.05 FWE corrected at the cluster level with a height threshold of *p* < 0.001; Fig. 4, Table 2).

**Figure 4.**
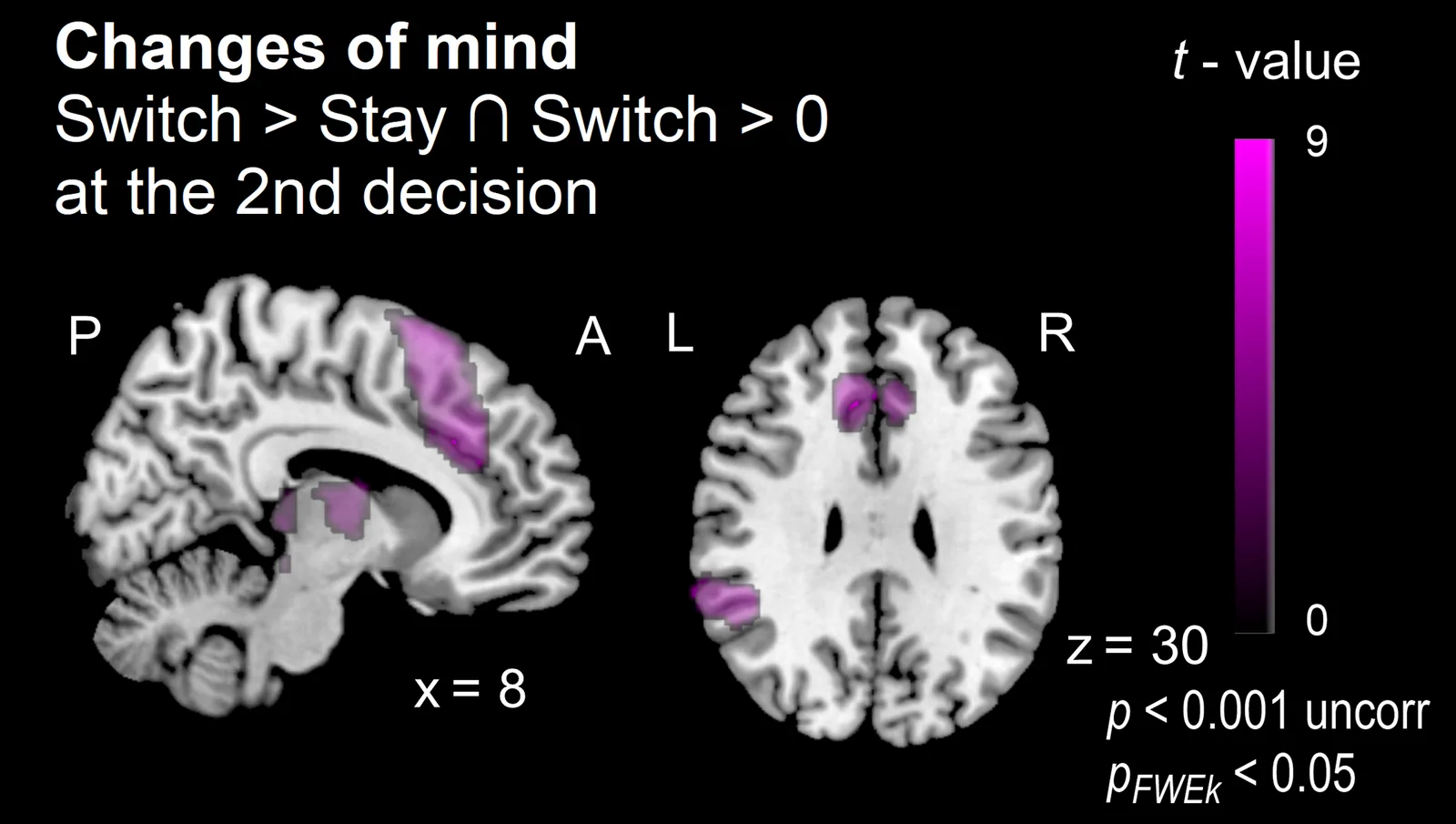
Neural activity related to changes of mind. We compared the neural activation when subjects changed their initial decision with the activation during stay trials at the second decision (switch > stay ∩ switch >0; *p* < 0.05 FWE corrected at the cluster level with a height threshold of *p* < 0.001). The color bar represents the color coding of the *t* value on the statistical map. The “*x*” beside the sagittal slice and “*z*” beside the axial slice denote the *x*- and *z*-coordinates of the displayed brain slices in MNI space, respectively. P, posterior; A, anterior; L, left; R, right. A control analysis addressing confounding factors is provided in Extended Data Figure 4-1.

10.1523/ENEURO.0104-25.2025.f4-1 Figure 4-1 **Behavioral correlations with switch events** This figure reports Pearson correlation coefficients between the second-decision switch event and each behavioral index. Continuous variables—stimulus difference, second-decision confidence, ΔConfidence, second-decision response time (RTs), and ΔRTs—were standardized to z-scores prior to analysis. Binary variables—second-decision correctness, error-correction, and switch—were coded as 1 = event and 0 = non-event. Here, “error-correction” refers to trials in which the first decision was incorrect but the second decision was subsequently correct. Download Figure 4-1, DOCX file.

**Table 2.** MNI coordinates of neural substrates of changes of mind

Switch > Stay ∩ Switch >0 at the 2nd decision							
Cluster size (mm^3^)	MNI coordinates				Peak value		Region label
	*x*	*y*	*z*	L/R	*t* value	*p* value FWE-corr	
7,170	55	19	8	R	8.47	0.000	Inferior frontal gyrus
	38	22	−11	R	8.30	0.000	Inferior frontal gyrus, pars triangularis
	40	28	2	R	6.63	0.018	Inferior frontal gyrus, pars triangularis
21,440	10	30	30	R	8.04	0.000	Dorsal anterior cingulate cortex
	5	22	52	R	7.78	0.001	Superior frontal gyrus
	12	19	63	R	7.78	0.001	Superior frontal gyrus
13,150	47	−44	32	R	6.61	0.019	Inferior parietal lobule
	57	−41	32	R	6.52	0.025	Inferior parietal lobule
	49	−52	52	R	5.36	0.350	Inferior parietal lobule
3,240	−32	22	−9	L	6.51	0.025	Anterior insular cortex
	−32	28	0	L	6.17	0.062	Anterior insular cortex
	−40	17	−7	L	5.59	0.220	Insular cortex
11,490	12	9	13	R	5.53	0.249	Caudate
	−1	−24	4	L	5.10	0.540	Thalamus
	−5	−7	2	L	4.98	0.644	Thalamus
4,130	−45	−50	−42	L	5.12	0.524	Cerebellum crus
	−36	−52	−37	L	4.98	0.645	Cerebellum crus
	−38	−63	−31	L	4.98	0.647	Cerebellum crus

To ensure that the observed switch-related activations were not driven by observable behavioral indices from the second decision, we calculated correlation coefficients between switch trials and behavioral measures (Extended Data Fig. 4-1). These analyses confirmed that the neural activity associated with changes of mind could not be explained by measurable factors alone. Thus, while changes of mind engage neural activity in regions such as the medial PFC—where we observed confidence-related signals—the spatial distribution of this activity is distinct from that associated with initial decision confidence.

### Common brain regions between initial confidence and changes of mind

The different brain activity patterns corresponding to confidence in initial decisions and subsequent control were identified. However, behavioral results showed that there were relationships between initial confidence and subsequent control (Fig. 2). To precisely test the spatial relationships of these neural activities, we performed a conjunction analysis between neural activity associated with metacognitive monitoring and that associated with changes of mind. Through this analysis, we uncovered common brain regions in the pregenual anterior cingulate cortex (pgACC; *p* < 0.05 FWE corrected at the cluster level with a height threshold of *p* < 0.001; Fig. 5*A*,*B*; Table 3). This spatial overlap suggests that the pgACC may contribute to both metacognitive monitoring and subsequent control (Palomero-Gallagher et al., 2019).

**Figure 5.**
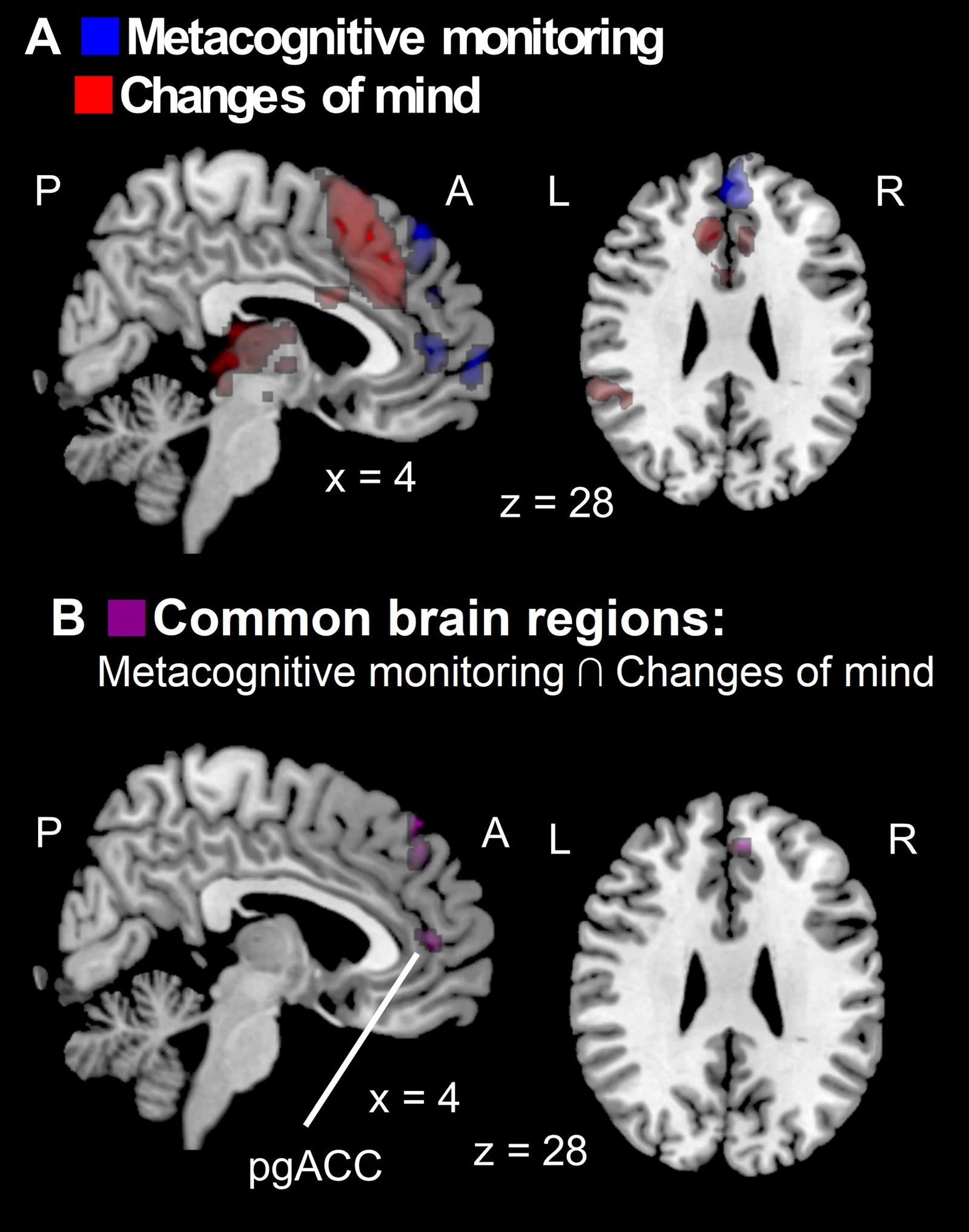
Spatial distributions of activated regions for two processes. ***A***, This panel illustrates the spatial distributions of neural activity of changes of mind (red) and initial confidence (blue). The overlapping colors indicate commonly activated areas. The “*x*” beside the sagittal slices and “*z*” beside the axial slices denote the *x*- and *z*-coordinates of the displayed brain slices in MNI space, respectively. P, posterior; A, anterior; L, left; R, right. ***B***, The panel shows the result of the conjunction analysis between neural activity associated with metacognitive monitoring and that associated with changes of mind. The regions with violet color represent the common activation between initial confidence and changes of mind (*p* < 0.05 FWE corrected at the cluster level with a height threshold of *p* < 0.001). Other conventions are the same as in ***A***.

**Table 3.** MNI coordinates of common brain regions between metacognitive monitoring and changes of mind

Common brain regions: metacognitive monitoring ∩ changes of mind							
Cluster size (mm^3^)	MNI coordinates				Peak value		Region label
	*x*	*y*	*z*	L/R	*t* value	*p* value FWE-corr	
910	−3	45	30	L	5.29	0.376	Pregenual anterior cingulate cortex
	−1	41	39	L	5.05	0.556	Pregenual anterior cingulate cortex
	5	43	52	R	4.53	0.927	Superior frontal gyrus

### Effective connectivity

A spatial overlap between metacognitive processes was identified in the medial PFC, but the functional interactions and information flow between the regions involved remained to be established. Finally, we performed the stochastic DCM analysis to explore how information is transmitted from monitoring to control processes at the neural level. Based on the present analysis, we prepared six DCM models in each hemisphere, including different directionality (forward, backward, and bidirectional) and mediation (mediated and direct) families among the amPFC, pgACC, and dACC. The results of the random-effects BMS at the family level showed that the model in which the pgACC mediated information flow between the amPFC and dACC was favored over the direct model (Fig. 6). In terms of directionality, bidirectional coupling within the specified network outperformed strictly forward and backward configurations. Finally, there was no evidence of laterality. Therefore, the best model in the present study was Model 5, indicating that the pgACC plays a central role in connecting metacognitive monitoring and control.

**Figure 6.**
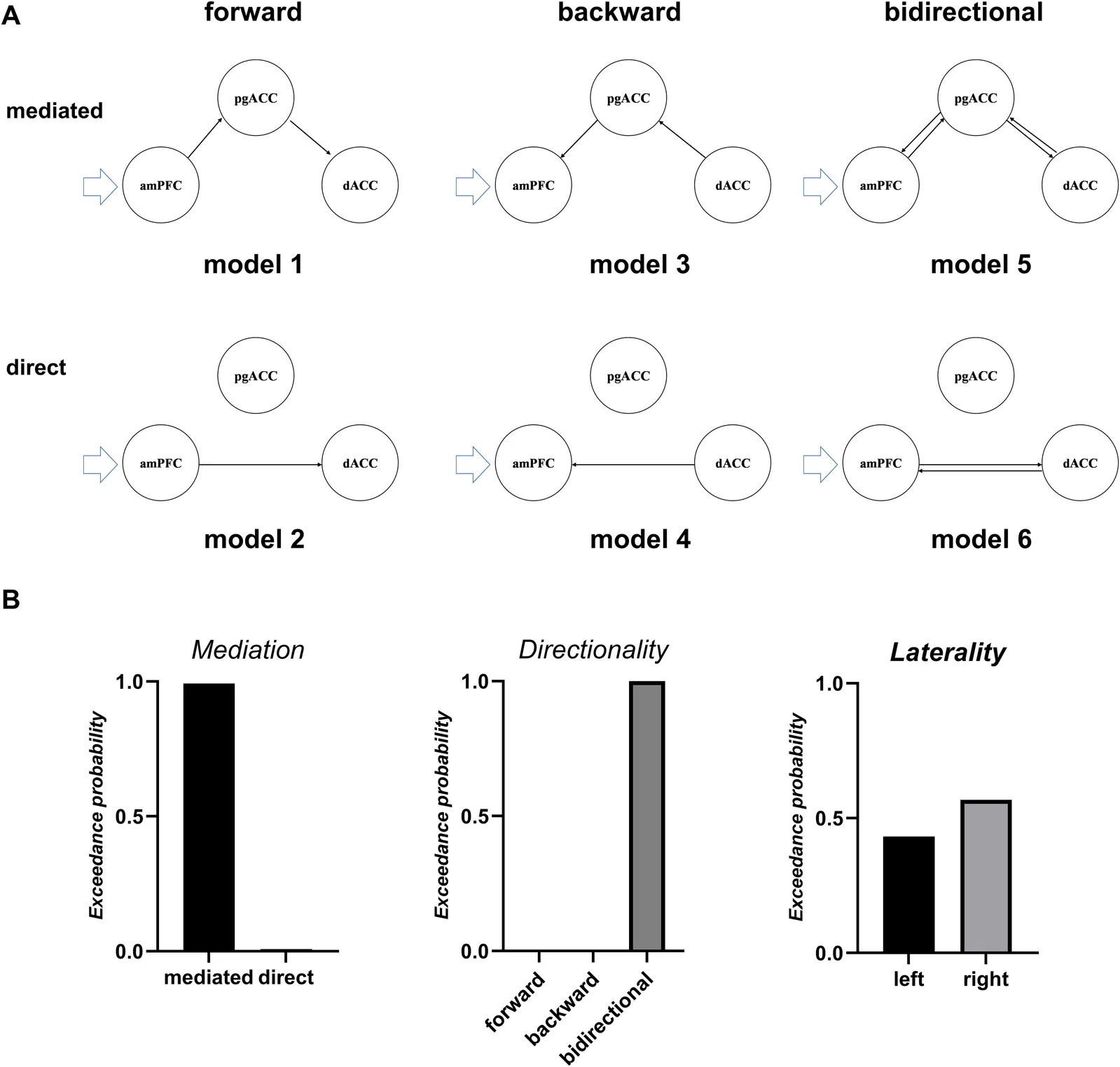
Effective connectivity in the medial PFC. These panels show the results of effective connectivity analysis among the amPFC, dACC, and pgACC. ***A***, Model structure of dynamic causal modeling. All models include the driving input of initial decisions into the amPFC. We tested six models in each hemisphere (a total of 12 models), including the mediated and direct models, distinct directionality (forward, backward, and bidirectional), and laterality (left and right sides). ***B***, DCM results. These panels display DCM results in the model family. All *y*-axes represent family-level exceedance probabilities. Values >0.95 indicate strong support for the corresponding family relative to the other families in the comparison. The results showed that Model 5 was the best among all models.

## Discussion

The present study investigated neural and behavioral mechanisms underlying metacognitive processes. By employing a sequential decision-making task with fMRI recording, this study found that confidence in initial decisions influenced the proportion of changes of mind in subsequent decision processes independently of perceptual stimuli. In fMRI analyses, the amPFC was associated with confidence in initial decisions, while the dACC showed significantly higher activity related to changes of mind in subsequent decision-making. Finally, stochastic DCM identified that the pgACC was involved in metacognitive monitoring and subsequent control and interacted with the amPFC and dACC.

### Confidence in single and sequential decision-making

While the neural substrates of confidence in isolated single-trial perceptual decisions have been well characterized (Fleming et al., 2010; Lei et al., 2020), how those confidence signals influence subsequent decisions remains unclear. To bridge this gap, we adopted a sequential decision-making paradigm to investigate whether—and how—confidence in initial decisions governs subsequent behavioral adjustments and underlying neural dynamics. In daily life, human behavior rarely concludes in a single decision; rather, each action tends to build upon the prior one, unfolding as a sequential ongoing process (Botvinick and Cohen, 2014), implying that metacognitive evaluation functions as a dynamic process evolving over successive decisions and monitoring episodes (Fleming and Daw, 2017).

### Role of confidence governing sequential behavior

This study demonstrates that confidence exerts a functional influence on subsequent decision processes (Fig. 2). While confidence covaries with objective accuracy—subjects report higher confidence for correct than for erroneous judgments (Butterfield and Mangels, 2003; Yeung and Summerfield, 2012; Boldt and Yeung, 2015; Harty et al., 2017; Kononowicz and van Wassenhove, 2019)—we found that initial confidence predicted changes of mind even after controlling for accuracy and sensory evidence (Fig. 2). Specifically, high confidence in an incorrect initial decision drove persistence of that error, whereas low confidence in an error facilitated its detection and correction. Conversely, high confidence in a correct decision enhanced its stability at the subsequent decision within the same trial. Thus, metacognitive evaluations—independent of external stimuli and objective accuracy—exhibit both facilitative effects (error detection and maintenance of correct choices) and biasing effects (confirmation bias; Nickerson, 1998; Yeung and Summerfield, 2012). Crucially, the duality of these metacognitive aspects might not be present if they are solely driven by external evidence. Together, the behavioral findings in the present study suggest that initial confidence plays a functionally significant role in shaping the subsequent cognitive processes.

### Metacognition in the medial prefrontal regions

In prior work, metacognitive monitoring and its neural substrates have often been dissociated anatomically between the lateral and medial PFC, with individual differences in metacognitive sensitivity relating to the lateral PFC and within-subject confidence judgments to the medial PFC (Fernandez-Duque et al., 2000; Fleming et al., 2010, 2012; McCurdy et al., 2013). Such an anatomical separation, however, might overstate the idea of a “single” locus for confidence representation. Indeed, confidence is unlikely to emerge from an isolated area but rather from interactions across a distributed network encompassing both the medial and lateral PFC as well as cingulate regions (Kiani and Shadlen, 2009; Bang and Fleming, 2018; Rouault et al., 2023).

Our results suggest that trial-by-trial confidence fluctuations are prominently—but not exclusively—represented in the medial PFC, specifically in the amPFC, whereas subsequent control signals were observed in the dACC. The medial PFC has been thought to be associated with self-referential processes (Kelley et al., 2002; Northoff and Bermpohl, 2004). These findings highlight a functional dissociation in terms of monitoring and control, rather than an exclusive anatomical segregation.

One possible reason for the apparent discrepancy with prior studies is that those approaches, using across-subject correlations between neural activity and metacognitive sensitivity, focus on the region that shows individual differences, but it is difficult to capture the full network of areas supporting confidence judgments. Taken together, these findings suggest that confidence emerges from a distributed metacognitive network, rather than being localized to any single region.

### Neural representations of subsequent control processes

We found that the dACC was involved in changes of mind, which were associated with subsequent control. The dACC showed higher neural activity in switch trials than in stay trials at the second decision. The activated regions also included the aIC, IPL, and IFG. The neural activity patterns in these regions for changes of mind are consistent with the findings of prior research on switching behavior (Mobbs et al., 2013; Philipp et al., 2013; Fleming et al., 2018). In foraging decision-making, the dACC supports such switching behavior (Hayden et al., 2011; Kolling et al., 2012; Mobbs et al., 2013). Furthermore, the activated regions, including the IPL and IFG, are associated with switching categories and responses in human behavior (Philipp et al., 2013). Thus, our neural findings on changes of mind support the findings of previous research (Hayden et al., 2011).

While our switch regressor robustly captures changes of mind, it exhibits a high overlap with an error correction index (Extended Data Fig. 4-1). This suggests that the observed neural activity may reflect both deliberate decision updating and spontaneous, error detection-driven correction. However, because our design employed a univariate GLM and the number of pure error correction trials was limited, we were unable to dissociate these subcomponents (Resulaj et al., 2009; Ullsperger et al., 2014). Future studies should adopt task paradigms that orthogonalize voluntary switches from corrective adjustments—either by interleaving forced-switch and free-switch conditions or by using multivariate/mixed-effects frameworks with sufficient trials—to disentangle the distinct neural mechanisms governing metacognitive monitoring, error detection, and adaptive decision updating.

### Network of connections with the pgACC

We hypothesized that if the neural activity conveys information from metacognitive monitoring to the control process, some brain regions should be commonly activated. A conjunction analysis between the neural activity associated with confidence in initial decisions and that associated with changes of mind revealed that the pgACC was the spatially common region. We further examined the effective connectivity among the three brain regions, including the amPFC, dACC, and pgACC, and found that the pgACC mediated between the other two brain regions (Fig. 6). The location of the pgACC between the amPFC and dACC indicates that the pgACC plays a role as an anatomical and functional hub (Palomero-Gallagher et al., 2019). These findings demonstrate that the pgACC can serve as a hub between metacognitive monitoring and subsequent control processes.

What effective connectivity endows the pgACC with the role of a mediating hub in the medial PFC? The pgACC has been thought to mediate the function of cost and benefit integration (Amemori and Graybiel, 2012; Klein-Flügge et al., 2022). This suggests that the pgACC receives information on costs and benefits from different sources and integrates them in the service of subsequent decision-making. It is even present in the rodents' medial frontal lobes, suggesting a phylogenetically old origin (Öngür and Price, 2000; Passingham and Wise, 2012). In line with this, the pgACC is situated in a strategically important location in the network of anatomical connections, which are linked with the areas related to the cost information (dACC), those implicated in the reward value (ventromedial PFC), and those related to metacognition (amPFC) (Passingham and Wise, 2012; Passingham, 2021).

We modeled effective connectivity among the amPFC, dACC, and pgACC using a stochastic DCM framework, directly comparing mediation models against direct models. Model selection via exceedance probabilities decisively favored the mediation model, indicating that the pgACC serves as a hub, linking monitoring to control signals. However, this inference is necessarily constrained by our predefined model space. For instance, we did not evaluate alternative routes or additional nodes known to contribute to confidence judgments. Moreover, our paradigm cannot distinguish the neural mechanisms in the dACC underlying voluntary changes of mind from those underlying externally instructed switching, a distinction that would require paradigms specifically tailored to separate these mechanisms. Despite these limitations, the present study provides a demonstration of the role of the pgACC in linking metacognitive monitoring and control within the medial PFC, and future work should both expand the model space and employ specialized experimental designs to map the metacognitive network.

In conclusion, the present study addresses behavioral and neural mechanisms linking metacognitive monitoring to subsequent control processes. Our results showed that confidence in the decision plays a functional role in influencing changes of mind in subsequent decision-making. Furthermore, a neural signature of confidence in the initial decision was reflected in the amPFC, while the dACC was involved in changes of mind, and the pgACC was a key brain region for connecting them. Thus, we propose that the pgACC plays a crucial role in linking metacognitive monitoring to control in the medial PFC.

## Synthesis

Reviewing Editor: Alexander Soutschek, Universitatsklinikum Wurzburg Decisions are customarily a result of the Reviewing Editor and the peer reviewers coming together and discussing their recommendations until a consensus is reached. When revisions are invited, a fact-based synthesis statement explaining their decision and outlining what is needed to prepare a revision will be listed below. The following reviewer(s) agreed to reveal their identity: Sabina Gherman. Note: If this manuscript was transferred from JNeurosci and a decision was made to accept the manuscript without peer review, a synthesis may not be available.

Reviewer #1

I reviewed the previous version of this manuscript and appreciate the substantial revisions authors have made to address earlier concerns, which I believe have improved the clarity and overall quality of the paper.

I have only one remaining point of clarification regarding the methodology for the stochastic DCM approach assessing connectivity among the amPFC/pgACC/dACC regions. The input to the C term (capturing task events that drive activity) is ambiguous in the text - the authors write "decision and confidence rating regressors were defined to capture trial-by-trial task inputs" (line 350), from which I couldn't figure out if they corresponded to event-related regressors or to parametric modulators (e.g., trial-by-trial confidence values), or both. I assume they are not parametric modulators (as those would be more appropriate for a modulatory B term), but a brief clarification would help.

Reviewer #2

The authors investigate the BOLD correlates of confidence and change-of-minds in a sequential perceptual decision-making task. Participants had to judge which of two circles was the largest and report their choice on a graded scale from confident it was left to confident it was right (the middle of the scale corresponded to low confidence). Then participants viewed the same stimulus again and re-reported their choice and confidence. There were four circle size differences corresponding to four levels of difficulty. Choice accuracy was better for the second decision, and change-of-minds depended on confidence, stimulus difficulty, and the accuracy of the first choice. At the neural level, there was an overlap between neural correlates of confidence after the first decision and of changes-of-mind after the second decision. The authors also performed some connectivity analyses. The authors interpret that confidence in the initial decision influences the proportion of change-of-minds independently from perceptual evidence. They conclude that the amPFC is associated with confidence in initial decisions, dACC with changes of mind, and pgACC with both metacognitive monitoring and subsequent control processes.

I had reviewed this manuscript for a different journal. I am happy to see that the authors have taken into account many of my comments:

- Interaction effects have been added (but discarded through model comparison).

- Response times regressors have been added to better attribute BOLD variability to confidence.

- The absence of significant clusters of activity related to stimulus differences has been reported.

- The authors have verified that the correlation between regressors is low enough.

- The authors abandoned the claims on external vs. internal components of confidence.

The authors have kept the median split of confidence and the ANOVAs instead of mixed models. I guess that is ok.

I still wonder how well the authors can dissociate activity in the second decision from activity in the first decision with no variance in the timing between the decisions. And I still think that there are a lot of variables involved and that the authors should discuss whether or not some findings can be explained by other variables.

For instance, the authors have one regressor for stay vs. switch on the second decision, but I guess that this regressor is correlated with -for example- the confidence in the second decision. So are we really looking at changes-of-mind in Fig.4 or just a lower confidence in the second decision because of the switch? Or activity from the first decision, but that could not be isolated by the experimental design? I think this is ok as long as it is discussed a little further than what has been done. More generally, I think a little tone-down of claims like:

'trial-by-trial fluctuations of confidence signals were most significantly represented in the medial PFC, specifically the amPFC, while the subsequent control process engaged the dACC' (590 - 592)

## Author Response

RESPONSE TO REVIEWER COMMENTS Dear Dr. Soutschek, Editor, and Reviewers, We sincerely appreciate your careful evaluation of our manuscript entitled "Neural mechanisms underlying metacognitive processes in sequential decision-making".

Your insightful and consensus-based synthesis, along with constructive suggestions, have been invaluable in helping us strengthen the rigor, clarity, and overall impact of our work.

In this revision, all changes in the main text have been marked with bold and underlined for your convenience. A clean version is also provided for potential publication.

Below, we provide detailed point-by-point responses to each of your comments, along with a summary of the corresponding changes made to the manuscript.

Reviewer #1:

I reviewed the previous version of this manuscript and appreciate the substantial revisions authors have made to address earlier concerns, which I believe have improved the clarity and overall quality of the paper.

Comment #1:

I have only one remaining point of clarification regarding the methodology for the stochastic DCM approach assessing connectivity among the amPFC/pgACC/dACC regions. The input to the C term (capturing task events that drive activity) is ambiguous in the text - the authors write "decision and confidence rating regressors were defined to capture trial-by-trial task inputs" (line 350), from which I couldn't figure out if they corresponded to event-related regressors or to parametric modulators (e.g., trial-by-trial confidence values), or both. I assume they are not parametric modulators (as those would be more appropriate for a modulatory B term), but a brief clarification would help.

Response #1:

Thank you for highlighting this point of ambiguity. We have now clarified the text in the revised Methods section (lines 349-353) to read: "One event regressor representing the first decision and confidence rating phase was used as the driving input (C matrix). The two parametric modulators from the first-level GLM-stimulus difference and confidence-were used to estimate neural time series but were not included in the C matrix. " In summary, the C matrix includes only the event-related regressor, and no parametric modulators were entered into it.

Reviewer #2:

The authors investigate the BOLD correlates of confidence and change-of-minds in a sequential perceptual decision-making task. Participants had to judge which of two circles was the largest and report their choice on a graded scale from confident it was left to confident it was right (the middle of the scale corresponded to low confidence). Then participants viewed the same stimulus again and re-reported their choice and confidence. There were four circle size differences corresponding to four levels of difficulty. Choice accuracy was better for the second decision, and change-of-minds depended on confidence, stimulus difficulty, and the accuracy of the first choice. At the neural level, there was an overlap between neural correlates of confidence after the first decision and of changes-of-mind after the second decision. The authors also performed some connectivity analyses. The authors interpret that confidence in the initial decision influences the proportion of change-of-minds independently from perceptual evidence. They conclude that the amPFC is associated with confidence in initial decisions, dACC with changes of mind, and pgACC with both metacognitive monitoring and subsequent control processes.

I had reviewed this manuscript for a different journal. I am happy to see that the authors have taken into account many of my comments:

- Interaction effects have been added (but discarded through model comparison).

- Response times regressors have been added to better attribute BOLD variability to confidence.

- The absence of significant clusters of activity related to stimulus differences has been reported.

- The authors have verified that the correlation between regressors is low enough.

- The authors abandoned the claims on external vs. internal components of confidence.

The authors have kept the median split of confidence and the ANOVAs instead of mixed models. I guess that is ok.

Comment #1:

I still wonder how well the authors can dissociate activity in the second decision from activity in the first decision with no variance in the timing between the decisions. And I still think that there are a lot of variables involved and that the authors should discuss whether or not some findings can be explained by other variables.

Response #1:

Thank you for raising this important concern. Although the task includes sequential decisions with a fixed inter-decision interval, our GLM design incorporates several features that allow us to dissociate neural activity related to the first and second decisions:

1. Distinct regressors and timing:

The first decision and confidence‐rating phase was modeled as a 2-second event regressor, time-locked to the onset of the VAS display. The second decision phase (both switch and stay trials) was modeled as a zero‐duration regressor at the subject's actual response time during the second decision.

2. Empirical response time variability (RT₂):

Switch trial RTs in the second decision phase exhibited considerable variability: mean = 0.625 s (SD = 0.212 s), range = 0.306 - 1.140 s.

When combined with the fixed 1.5 s inter‐decision interval and 1 s of second‐evidence presentation, this variability resulted in an average onset of second decisions ~3.2 s after the first event, providing meaningful temporal separation.

3. Distinct HRF profiles:

The different event durations (2 s for first‐decision vs. 0 s for second‐decision) yield distinct HRF‐convolved regressors (showing different amplitudes of each regressor), further supporting their separability in the GLM.

4. Low inter-regressor correlations:

We computed pairwise correlations among all regressors and confirmed that the highest absolute correlation was < 0.30, indicating low multicollinearity.

Together, these design features-empirical jitter in RT₂, distinct regressor durations and timing, and low correlation among regressors-support the dissociation of neural signals associated with the first and second decisions.

Comment #2:

For instance, the authors have one regressor for stay vs. switch on the second decision, but I guess that this regressor is correlated with -for example- the confidence in the second decision. So are we really looking at changes-of-mind in Fig.4 or just a lower confidence in the second decision because of the switch? Or activity from the first decision, but that could not be isolated by the experimental design? I think this is ok as long as it is discussed a little further than what has been done.

Response #2:

Thank you for highlighting the potential influence of behavioral confounds. To ensure that our switch regressor reflects genuine changes-of-mind signals rather than being driven by differences in second-decision confidence or other behavioral indices, we conducted the following control analyses:

1. Behavioral correlations: We computed Pearson correlations between the binary switch regressor and various second decision metrics, including stimulus difference, confidence₂, Δconfidence, RT, ΔRT, and correctness₂. All correlations were weak (|r| < 0.27; see Extended Data Figure 4-1), indicating that no single behavioral variable explains the switch effect.

However, we observed a strong correlation between error correction and switching (r > 0.7), suggesting that error correction is a substantial component of changes of mind. This supports the interpretation that the neural activity in Figure 4 reflects broader changes-of-mind processes, which encompass-but are not limited to-error correction.

2. Revised Results section (lines 509-513):

To confirm that the switch related signal was not driven by measurable second-decision variables, we included this correlation analysis in the Results: "To ensure that the observed switch-related activations were not driven by observable behavioral indices from the second decision, we calculated correlation coefficients between switch trials and behavioral measures (see Extended Data Figure 4-1). These analyses confirmed that the neural activity associated with changes of mind could not be explained by measurable factors alone." 3. Expanded Discussion (lines 622-633):

The revised statement read as follows: "While our switch regressor robustly captures changes of mind, it exhibits a high overlap with an error correction index (see Extended Data Figure 4-1). This suggests that the observed neural activity may reflect both deliberate decision updating and spontaneous, error-detection driven correction. However, because our design employed a univariate GLM and the number of pure error-correction trials was limited, we were unable to dissociate these subcomponents (Resulaj et al., 2009; Ullsperger et al., 2014). Future studies should adopt task paradigms that orthogonalize voluntary switches from corrective adjustments-either by interleaving forced-switch and free‐switch conditions, or by using multivariate/mixed‐effects frameworks with sufficient trials-to disentangle the distinct neural mechanisms governing metacognitive monitoring, error detection, and adaptive decision updating. " Taken together, these additions clarify that the neural activity associated with switching cannot be explained solely by second-decision confidence, stimulus difficulty, or related behavioral metrics. We hope this explanation fully addresses your concern.

Comment #3:

More generally, I think a little tone-down of claims like: 'trial-by-trial fluctuations of confidence signals were most significantly represented in the medial PFC, specifically the amPFC, while the subsequent control process engaged the dACC' (590 - 592) Response #3:

Thank you for your suggestion. We have revised the wording to present a more cautious interpretation of our findings. The revised manuscript (lines 596-598) now reads: "Our results suggest that trial by trial confidence fluctuations are prominently-but not exclusively-represented in the medial PFC, specifically in the amPFC, whereas subsequent control signals were observed in the dACC." This change preserves the main interpretation while avoiding overstatement and better reflects the nuances of our data.

We appreciate your thoughtful feedback throughout the review process. We believe that these revisions have improved the clarity, rigor, and balance of our manuscript. Thank you once again, and we look forward to your final evaluation.

Sincerely, Yoshinori Nanjo, on behalf of all authors
