# Supplementary material for: Neural Mechanisms Underlying Metacognitive Processes in Sequential Decision-Making
Source: eNeuro. 2026 Oct 28;13(10):ENEURO.0104-25.2025. doi: 10.1523/ENEURO.0104-25.2025 (PMC13718379; doi:10.1523/ENEURO.0104-25.2025)
Supplement: Supplementary file 1 [file eneuro-13-ENEURO.0104-25.2025-s002.docx]

**Figure 2-1. Correlation matrix among confidence, stimulus, and correctness**

To assess multicollinearity in our behavioral data analyses on changes of mind, this figure displays correlation coefficients among confidence, stimulus, and correctness. All off-diagonal correlations were less than 0.30, indicating negligible collinearity.
