# Supplementary material for: Neural Mechanisms Underlying Metacognitive Processes in Sequential Decision-Making
Source: eNeuro. 2026 Oct 28;13(10):ENEURO.0104-25.2025. doi: 10.1523/ENEURO.0104-25.2025 (PMC13718379; doi:10.1523/ENEURO.0104-25.2025)
Supplement: Supplementary file 2 [file eneuro-13-ENEURO.0104-25.2025-s003.docx]

**Figure 2-2. VIF in behavioral data analyses**

To assess the degree of multicollinearity, we calculated the variance inflation factor (VIF). This figure shows that all VIFs are below 5.00, indicating that multicollinearity is negligible.
