# Supplementary material for: Neural Mechanisms Underlying Metacognitive Processes in Sequential Decision-Making
Source: eNeuro. 2026 Oct 28;13(10):ENEURO.0104-25.2025. doi: 10.1523/ENEURO.0104-25.2025 (PMC13718379; doi:10.1523/ENEURO.0104-25.2025)
Supplement: Supplementary file 4 [file eneuro-13-ENEURO.0104-25.2025-s006.docx]

**Figure 4-1. Behavioral correlations with switch events**

This figure reports Pearson correlation coefficients between the second‐decision switch event and each behavioral index. Continuous variables—stimulus difference, second‐decision confidence, ΔConfidence, second‐decision response time (RTs), and ΔRTs—were standardized to z‐scores prior to analysis. Binary variables—second‐decision correctness, error‐correction, and switch—were coded as 1 = event and 0 = non‑event. Here, “error‑correction” refers to trials in which the first decision was incorrect but the second decision was subsequently correct.
