# Supplementary material for: Neural Mechanisms Underlying Metacognitive Processes in Sequential Decision-Making
Source: eNeuro. 2026 Oct 28;13(10):ENEURO.0104-25.2025. doi: 10.1523/ENEURO.0104-25.2025 (PMC13718379; doi:10.1523/ENEURO.0104-25.2025)
Supplement: Supplementary file 5 [file eneuro-13-ENEURO.0104-25.2025-s005.docx]

**Figure 3-2. MNI coordinates of neural substrates of initial confidence in GLM2**
